# Energy metabolic dysfunction as a carcinogenic factor in cancer cells

**DOI:** 10.1186/s40169-016-0094-5

**Published:** 2016-04-06

**Authors:** Yongyan Sun, Zhenhua Shi, Huiyong Lian, Peng Cai

**Affiliations:** Physical Environment Laboratory, Institute of Urban Environment, Chinese Academy of Sciences, 1799 Jimei Avenue, Xiamen, 361021 People’s Republic of China; University of Chinese Academy of Sciences, 19 Yuquan Road, Beijing, 100049 People’s Republic of China; Environmental Bioelectrochemistry Center, Fujian Agriculture and Forestry University, Fuzhou, 350002 Fujian People’s Republic of China

## Abstract

Cancer, as a leading cause of death, has attracted enormous public attention. Reprogramming of cellular energy metabolism is deemed to be one of the principal hallmarks of cancer. In this article, we reviewed the mutual relationships among environmental pollution factors, energy metabolic dysfunction, and various cancers. We found that most environmental pollution factors could induce cancers mainly by disturbing the energy metabolism. By triggering microenvironment alteration, energy metabolic dysfunction can be treated as a factor in carcinogenesis. Thus, we put forward that energy metabolism might be as a key point for studying carcinogenesis and tumor development to propose new methods for cancer prevention and therapy.

## Introduction

To date, cancer has become a major public health problem and has caused global-scale morbidity and mortality in modern society. With approximately 14 million new cases and 8 million cancer-related deaths in 2012, cancer affects populations in all countries and all regions [1]. One of the main characteristics of cancer cells is limitless replication potential [2], which results in high energy requirement. Cancer cells also deregulate energy homeostasis [3]. Thus we cannot ignore the strong connection between cancers and energy metabolism, Accordingly, seeking new effective therapeutic methods for cancers from the perspective of energy metabolic dysfunction as a carcinogenesis factor could be possible.

Energy metabolism is one of the most basic characteristics of living organisms. It is associated with the progress of metabolic reactions catalyzed by a variety of enzymes. The mitochondria are crucial in energy metabolism [4]. Most of the cellular energy required for various biological functions is provided by the mitochondria through oxidative phosphorylation [5].

Carcinogenesis and energy metabolism can be both influenced by the environment. Growing evidence has demonstrated that several environmental factors, including physical, chemical, and biological environmental factors, can disturb cellular energy metabolism. When energy metabolic dysfunction occurs, the living cell microenvironment and surroundings encounter alterations, which may become conducive to cancer cell proliferation. Leading alterations include acidity [6] and interstitial fluid pressure [7] in the microenvironment, such alterations can promote risks of carcinogenesis. Besides this, hypoxic microenvironment is tightly correlated with cancer progression as well [8]. Therefore, environmental pollution factors can disturb energy homeostasis by triggering microenvironment alteration, thereby increasing carcinogenic risks.

In general, we briefly review the relationships among energy metabolism, cancers, tumor microenvironment, and environmental factors, to attempt to provide new perspectives on cancer prevention and treatment.

## Review

### Energy metabolism disorders in cancer cells

Since Warburg reported that tumor cells in living organisms were associated with abnormal energy utilization [9], this phenomenon has continuously attracted research attention. Warburg first noted the increased intake of glucose and lactate production even in the presence of oxygen (aerobic glycolysis) in tumor cells, subsequently this phenomenon was named the “Warburg effect”. The Warburg effect has been implicated in cell transformation, immortalization, and proliferation during tumorigenesis [10]. At present, most studies have demonstrated that energy metabolic dysfunction is one of the major features of cancer cells, and it can be driven by multiple factors, such as the effect of oncogenes, tumor suppressors, mitochondrial DNA (mtDNA) mutations, and signal pathways, etc. [11].

Several researches suggested that the altered metabolism with aerobic glycolysis was a feature of cancers rather than a cause [12]. In malignant melanoma cells, the oncogene BRAF upholds the activity of glycolysis and therefore the addiction to glycolysis becomes an addiction to BRAF [13]. Furthermore, tumor suppressor p53 has been proven to be related to several energy metabolic pathways in cancer cells, such as the tricarboxylic acid cycle (TCA cycle), glucose transportation, and glycolysis [14]. Growing evidence also indicated that mtDNA mutations can increase the reactive oxygen species (ROS) production and contribute to tumorigenesis through the inhibition of oxidative phosphorylation [15]. What’s more, several signal pathways involved in energy metabolism in cancer cells show abnormal conditions in compared with those in normal cells. The Akt-signal pathway mediates multiple cell activities, including cell cycle, apoptosis, and glycogen synthesis, which are all perturbed in cancer cells. The Akt-signal pathway has well been reported to be involved in the regulation of intracellular glucose levels favoring hexokinase-mitochondria interaction [16, 17]. In particular, PI3 K–Akt–mTOR signaling, plays an important role in coordinating metabolism and promoting cell survival, and the specific contributions of Akt hyperactivation to oncogenes have been attributed to its fundamental roles in cellular energy metabolism of inhibiting apoptosis, increasing cell proliferation, and accelerating oncogenic mutation rates [18].

Aside from the Warburg effect found in cancer cells, other abnormal energy metabolic alterations have been studied in recent years. The lower ATP generating efficiency of glycolysis in comparison with oxidative phosphorylation makes the cancer cells with more glucose uptake. Therefore, glucose transporters, which are transmembrane proteins in a series, were reported to be upregulated in various cancers [19, 20]. Glutaminolysis alteration also occurs in cancer cells [21]. Increasing evidence indicated that elevated expression of glutaminase enhanced glucose utilization by glutaminolysis in prostate cancer [22], and the function of glutaminolysis regulation has been discussed by scientists [23]. Several reports have asserted that glutamine can be involved in multiple energy metabolism-related processes, including TCA cycle, gluconeogenic precursor, and lipogenic precursor, etc. therefore cancer cells benefit from the activation of glutaminolysis, which can also promote the pentose phosphate pathway to generate more NADPH. Consequently, NADPH can generate reduced glutathione (GSH) and decrease the ROS levels to protect the cancer cells from excessive oxidative stress [14].

However, a recent scientific report has proposed an evolutionary theory that challenges the current understanding on cancers, suggesting that cancers are the generated products of tissue microenvironment alteration [24]. Microenvironment alteration can be tightly linked with cell energy metabolic dysfunction. Some oncogenic mutations gain the upper hand and develop into malignant tumors because the ‘ecosystem’ of a tissue is altered in abnormal situations [24]. The said report provides new perspectives on the mechanisms of carninogenesis and for better therapetic methods for cancers. Given the potential relationship between microenvironment and cancers, we first summarize the driving environmental factors of cancer-linked energy metabolic dysfunction.

### Environmental factors contributing to energy metabolism dysfunction

Carcinogenic environmental factors are divided into three types according to their properties, namely, physical, chemical, and biological factors. Among the physical factors, the most familiar factor is light, especially ultraviolet, which promotes risks of cancer generation by inducing gene mutations and disturbing glycolysis [25]. Thus, energy metabolism might be altered through the exposure to ultraviolet, and such alteration might be relate to cancer generation. Electricity is another physical environmental factor linked to energy dysfunction. Ionizing radiation can induce endogenously generated ROS. High intra-mitochondrial ROS levels damage the mt DNA and those mutations can affect the epigenetic control mechanisms of the nuclear DNA by decreasing the activity of methyltransferases, thus causing global DNA hypomethylation [26]. These changes might increase the risks of mutation among energy metabolism-related genes and activate the epigenetic regulation of cellular energy homeostasis. Our recent study on the effect of electromagnetic field on energy metabolism of *Caenorhabditis elegans* has shown an upregulation of the gene expression of glycolysis-related enzymes at mRNA levels [27], suggesting that the relationships between this alteration and carcinogenic risks should be further investigated.

With regard to the diversified effective mechanisms of chemical factors on energy metabolism, one of the traditional perspectives implied that cancers are associated with genetic toxicity. Several chemicals can directly or indirectly result in DNA damage (mtDNA or nuclear DNA damage) [28, 29]. For example, chemicals may bind to DNA to initiate a complexity or disorder in DNA repair; as such, energy-related cell activities, such as cell proliferation, cannot be controlled. Moreover, growing evidence has shown that the mutation of ALDH2 involved in the oxidative pathway of alcohol metabolism can promote hepatocarcinogenesis in murine [30]. Polychlorinated biphenyls, which are potent inducers of toxic ROS, have been reported to be capable of inducing DNA damage and activating oxidative stress responses [31]. Besides the mutational events in chemical carcinogenesis, epigenetic alterations also affect the metabolism of cells and may be crucial for the development of cancer cells [32, 33]. Chemicals can also cause tumors by mechanisms other than directly damaging DNA. Mounting evidence indicates that the disruption of epigenetic balance can lead to diseases, including cancers. The contributions of various environmental, non-genotoxic carcinogens, such as polycyclic aromatic hydrocarbons, benzene, and *N*-nitrosamines, to induce methylome changes associated with oncogenic progression have been shown in lung, colorectal, and liver oncogenesis, as well as leukemogenesis [34, 35]. Metals such as lead, nickel, cobalt, and mercury have been reported to disrupt DNA repair, with nickel affecting epigenetic histone modification and causing defective telomere maintenance [36, 37]. A number of key metabolites, including SAM, acetyl-CoA, NAD(+), and ATP, serve as essential co-factors for many, perhaps most, epigenetic enzymes that regulate DNA methylation, post-translational histone modifications, and nucleosome position [38]. Thus, chemical factors inducing the abnormal levels of energy metabolites can increase the carcinogenic risks. For instance, benzene poisoning increased the content level of lipid peroxidation products and mitochondrial energetic activities [39], and high peroxidation products level was reported to induce epigenetic alterations in human carcinogenesis [40].

For the biological factors that can affect the mentioned energy metabolic levels, we mainly focused on the influence of microorganisms. Viral, bacterial and fungal infections can all affect the energy utilization of the body and perturb the balance of cell metabolic activities [41–43]. The relationship between infection and cancers is a perennial object of study. Among these researches and clinical cases, Rous et al. first reported the biological and chemical pathogenic effects on cancers, and demonstrated the connection between cancer susceptibility and body alteration induced by infection [44]. From the in-depth study on *Helicobacter pylori* (HP) infection, several findings have suggested that HP infection is linked to the pathogenesis of gastric cancer to some extent, and a key energy-metabolism related enzyme has been investigated simultaneously [45]. HP infection can induce instability in mtDNA [46], and mutations in mtDNA can activate the mitochondrial oxidative phosphorylation pathway [47], further influencing energy generation and utilization. Additionally, human papillomavirus (HPV) infection has been proven correlated with colorectal cancer [48], cervical cancer [49], and head and neck cancer [50]. Biological environmental factors, including hepatitis B and hepatitis C viruses (HBV and HCV), are considered among the main causes of hepatocellular carcinoma, which should not be ignored as well [51]. Hence, the potential of energy metabolic dysfunction to increase carcinogenic risks induced by biological factors need to be examined further.

Aside from the environmental factors, other aspects, particularly individual subjective psychological factors, can take participate in the dysfunctional energy metabolism related to cancers [52, 53].

### Environmental factors contributing to carcinogenesis and cancer progression by microenvironment disruption

In carcinogenesis and cancer development, carcinogenic factors and co-carcinogenic factors are highly correlated with the human living environment and activities. Complex multi-functional environmental factors can alter the concentration and constituents of tissue metabolites. The microenvironment of living cells can be a potential driving factor of carcinogenesis.

Numerous researchers have concluded that tissue microenvironment is an important determinant of carcinogenesis. One of the contributing factors to cancer cell adaptation is a hypoxic environment [54]. Hypoxia is associated with increased metastatic potential, development of resistance to therapies, and poor prognosis [8]. Hypoxic stress can activate the HIF-signal pathway, which presents a strong relationship with the energy metabolic process, especially in regulating the triosephosphate isomerase expression [55]. Energy metabolic dysfunction can promote the establishment of a hypoxic microenvironment, particularly through abnormal mitochondrial aerobic respiration and increased rate of glycolysis. An acidic tissue microenvironment is also correlated with energy metabolic dysfunction, mainly because a high rate of glycolysis generates a large amount of lactic acid, which promotes the generation of acidic tissue microenvironment [56]. Another contributing factor is elevated interstitial fluid pressure (IFP). The mechanism for elevated IFP was reported to be the dysfunction of the lymphatic system [57]; such dysfunction can help cancer cells escape from the attack of functional lymphatics and create conducive conditions for various metabolites and cell growth factors and cytokines [58].

To emphasize the importance and complexity of the microenvironment to cancers, we discuss how environmental factors, such as physical, chemical and biological factors, alter the microenvironment. For physical factors, several scientists have reported that radiation can induce aerobic glycolysis through ROS generation [59]. Extracellular acidification of the microenvironment has been ascribed to lactate secretion from glycolysis [60]. Therefore, radiation can act on the tumor microenvironment by regulating glycolysis. Chemical environmental factors, including various natural chemical metabolites and synthetic chemicals, affect cellular microenvironments in different ways. One of the approaches disruptive chemicals contribute to the tumor microenvironment is perturbing energy homeostasis. For example, chemicals affect the gene expression of enzymes involved in energy metabolism, thereby promoting the rapid growth and proliferation of cells [61]. The persistently high level of energy utilization can establish a hypoxia microenvironment for the initiation and development of cancers [62].

## Conclusions

**Fig. 1:**
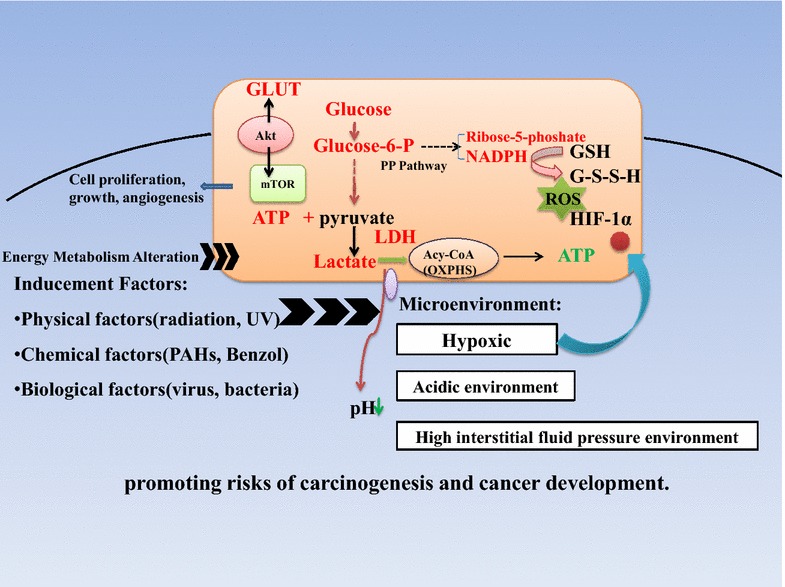
Schematic of energy metabolism dysfunction promoting carcinogenic risks and cancer development. Different types of environmental factors (physical, chemical and biological factors) could act on cancer cells and the tumor microenvironment. Cancer cells usually show aerobic glycolysis and abnormal signal pathway regulations. Pentose phosphate pathway can be upregulated to increasing the amount of NADPH and ribose-5-phoshate, which are used for energy generation and DNA replication, as well as counteracting proportion of ROS for protecting cancer cells from oxidative stress. The inhibition of oxidative phosphorylation at the same time can result in the accumulation of lactate and the acid microenvironment. Moreover, several signal pathways, such as the Akt-mTOR signal pathway and the HIF signal pathway, are involved in the dysfunction of energy metabolism and can co-active a hypoxic tumor microenvironment

**Fig. 2:**
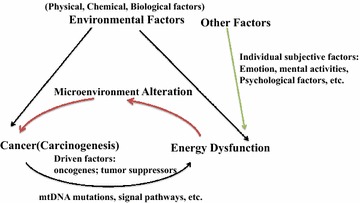
Possible relationships between environmental factors, cancers (mainly carcinogenesis), and energy metabolism. One of the characteristics of cancer cells is energy dysfunction, which can be driven by multiple factors, such as oncogenes, and tumor suppressors, etc. Energy dysfunction can also directly or indirectly contribute to cancers as a carcinogenic factor, probably by affecting the microenvironment. Environmental factors might be involved in carcinogenesis mediated by energy dysfunction, or directly involved on the progress of cancer initiation. Other factors, such as individual subjective factors, can also contribute to energy dysfunction

Considering the significant role energy metabolism in organisms, energy metabolic dysfunction should be given substantial attention, particularly on its connection to human cancers. A sketch of the energy metabolic dysfunction promoting the risks of carcinogenesis and cancer development is shown in Fig. 1. The alteration in energy utilization and generation of cancer cells, that is, the occurrence of aerobic glycolysis and disordered mitochondrial oxidative phosphorylation can provide a conductive condition for carcinogenesis and tumor development. Other alterations, namely glutaminolysis, ROS level, and signal pathways, can be closely associated with energy metabolism. The tumor microenvironment which usually shows the features of hypoxia, accumulation of acidic metabolites and interstitial fluid pressure, provides a suitable microenvironment for uncontrollable cell proliferation and increases the risks of cancer generation. Energy metabolism alteration can be related to environmental factors, which we classified into three categories, namely physical, chemical, and biological factors. The possible relationships among environmental factors, cancers (mainly carcinogenesis), and energy metabolism are presented in Fig. 2. In the figure, we emphasize that energy metabolic dysfunction can directly or indirectly contribute to cancers as a carcinogenic factor, probably by affecting the microenvironment. Furthermore, other factors, such as individual subjective factors, and psychological factors, can be involved in energy metabolic dysfunction. We propose that owing to the important role of energy metabolism dysfunction in promoting cancer risks, energy metabolic dysfunction as a carcinogenic factor cannot be ignored.

## References

[1] Cancer IAfRo (2014) World cancer report 2014. WHO, Geneva

[2] Marahatta SB, Sharma N, Koju R, Makaju RK, Petmitr P, Petmitr S (2005). Cancer: determinants and progression. Nepal Med Coll J.

[3] Ashrafian H, Ahmed K, Rowland SP, Patel VM, Gooderham NJ, Holmes E (2011). Metabolic surgery and cancer: protective effects of bariatric procedures. Cancer.

[4] Wallace DC (2012). Mitochondria and cancer. Nat Rev Cancer.

[5] Brookes PS, Yoon Y, Robotham JL, Anders MW, Sheu SS (2004). Calcium, ATP, and ROS: a mitochondrial love-hate triangle. Am J Physiol Cell Physiol.

[6] Navratilova J, Hankeova T, Benes P, Smarda J (2013). Acidic pH of tumor microenvironment enhances cytotoxicity of the disulfiram/Cu^2+^ complex to breast and colon cancer cells. Chemotherapy.

[7] Milosevic MF, Pintilie M, Hedley DW, Bristow RG, Wouters BG, Oza AM (2014). High tumor interstitial fluid pressure identifies cervical cancer patients with improved survival from radiotherapy plus cisplatin versus radiotherapy alone. Int J Cancer.

[8] Semenza GL (2015). The hypoxic tumor microenvironment: a driving force for breast cancer progression. Biochim Biophys Acta.

[9] Warburg O, Wind F, Negelein E (1927). The metabolism of tumors in the body. J Gen Physiol.

[10] Oliveira PF, Martins AD, Moreira AC, Cheng CY, Alves MG (2015). The Warburg effect revisited–lesson from the Sertoli cell. Med Res Rev.

[11] Kroemer G, Pouyssegur J (2008). Tumor cell metabolism: cancer’s Achilles’ heel. Cancer Cell.

[12] Harris AL (2002). Hypoxia—a key regulatory factor in tumour growth. Nat Rev Cancer.

[13] Hall A, Meyle KD, Lange MK, Klima M, Sanderhoff M, Dahl C (2013). Dysfunctional oxidative phosphorylation makes malignant melanoma cells addicted to glycolysis driven by the (V600E)BRAF oncogene. Oncotarget.

[14] Chen JQ, Russo J (2012). Dysregulation of glucose transport, glycolysis, TCA cycle and glutaminolysis by oncogenes and tumor suppressors in cancer cells. Biochim Biophys Acta.

[15] Petros JA, Baumann AK, Ruiz-Pesini E, Amin MB, Sun CQ, Hall J (2005). mtDNA mutations increase tumorigenicity in prostate cancer. Proc Natl Acad Sci USA.

[16] Robey RB, Hay N (2006). Mitochondrial hexokinases, novel mediators of the antiapoptotic effects of growth factors and Akt. Oncogene.

[17] Robey RB, Weisz J, Kuemmerle NB, Salzberg AC, Berg A, Brown DG (2015). Metabolic reprogramming and dysregulated metabolism: cause, consequence and/or enabler of environmental carcinogenesis?. Carcinogenesis.

[18] Lim HJ, Crowe P, Yang JL (2015). Current clinical regulation of PI3 K/PTEN/Akt/mTOR signalling in treatment of human cancer. J Cancer Res Clin Oncol.

[19] Krzeslak A, Wojcik-Krowiranda K, Forma E, Jozwiak P, Romanowicz H, Bienkiewicz A (2012). Expression of GLUT1 and GLUT3 glucose transporters in endometrial and breast cancers. Pathol Oncol Res: POR.

[20] Szablewski L (2013). Expression of glucose transporters in cancers. Biochim Biophys Acta.

[21] Friday E, Oliver R, Welbourne T, Turturro F (2011). Glutaminolysis and glycolysis regulation by troglitazone in breast cancer cells: relationship to mitochondrial membrane potential. J Cell Physiol.

[22] Pan T, Gao L, Wu G, Shen G, Xie S, Wen H (2015). Elevated expression of glutaminase confers glucose utilization via glutaminolysis in prostate cancer. Biochem Biophys Res Commun.

[23] Dang CV (2010). Glutaminolysis: supplying carbon or nitrogen or both for cancer cells?. Cell Cycle.

[24] Rozhok AI, DeGregori J (2015). Toward an evolutionary model of cancer: considering the mechanisms that govern the fate of somatic mutations. Proc Natl Acad Sci USA.

[25] Kricker A, Armstrong BK, McMichael AJ (1994). Skin cancer and ultraviolet. Nature.

[26] Szumiel I (2015). Ionizing radiation-induced oxidative stress, epigenetic changes and genomic instability: the pivotal role of mitochondria. Int J Radiat Biol.

[27] Shi Z, Yu H, Sun Y, Yang C, Lian H, Cai P (2015). The energy metabolism in caenorhabditis elegans under the extremely low-frequency electromagnetic field exposure. Sci Rep.

[28] Teitelbaum SL, Belpoggi F, Reinlib L (2015). Advancing research on endocrine disrupting chemicals in breast cancer: expert panel recommendations. Reprod Toxicol.

[29] Antwi SO, Eckert EC, Sabaque CV, Leof ER, Hawthorne KM, Bamlet WR (2015). Exposure to environmental chemicals and heavy metals, and risk of pancreatic cancer. Cancer Causes Control.

[30] Jin S, Chen J, Chen L, Histen G, Lin Z, Gross S (2015). ALDH2(E487 K) mutation increases protein turnover and promotes murine hepatocarcinogenesis. Proc Natl Acad Sci USA.

[31] Li R, Cao S, Dai J, Wang L, Li L, Wang Y (2014). Effect of caffeic acid derivatives on polychlorinated biphenyls induced hepatotoxicity in male mice. J Biomed Res.

[32] Tabish AM, Poels K, Hoet P, Godderis L (2012). Epigenetic factors in cancer risk: effect of chemical carcinogens on global DNA methylation pattern in human TK6 cells. PLoS ONE.

[33] Koturbash I, Beland FA, Pogribny IP (2011). Role of epigenetic events in chemical carcinogenesis–a justification for incorporating epigenetic evaluations in cancer risk assessment. Toxicol Mech Methods.

[34] Casey SC, Vaccari M, Al-Mulla F, Al-Temaimi R, Amedei A, Barcellos-Hoff MH (2015). The effect of environmental chemicals on the tumor microenvironment. Carcinogenesis.

[35] Pogribny IP, Beland FA (2013). DNA methylome alterations in chemical carcinogenesis. Cancer Lett.

[36] Arita A, Niu J, Qu Q, Zhao N, Ruan Y, Nadas A (2012). Global levels of histone modifications in peripheral blood mononuclear cells of subjects with exposure to nickel. Environ Health Perspect.

[37] Chervona Y, Arita A, Costa M (2012). Carcinogenic metals and the epigenome: understanding the effect of nickel, arsenic, and chromium. Metallomics.

[38] Donohoe DR, Bultman SJ (2012). Metaboloepigenetics: interrelationships between energy metabolism and epigenetic control of gene expression. J Cell Physiol.

[39] Trifonov Iu A, Turdyev AA, Tiunov LA, Ivanov VI, Voloshin SV (1989). Oxidative phosphorylation and lipid peroxidation of the liver mitochondria in rats after acute benzene poisoning. Gig Sanit.

[40] Ziech D, Franco R, Pappa A, Panayiotidis MI (2011). Reactive oxygen species (ROS)–induced genetic and epigenetic alterations in human carcinogenesis. Mutat Res.

[41] Decsi T, Koletzko B (2000). Effects of protein-energy malnutrition and human immunodeficiency virus-1 infection on essential fatty acid metabolism in children. Nutrition.

[42] Paton NI, Angus B, Chaowagul W, Simpson AJ, Suputtamongkol Y, Elia M (2001). Protein and energy metabolism in chronic bacterial infection: studies in melioidosis. Clin Sci (Lond).

[43] Staats CC, Kmetzsch L, Schrank A, Vainstein MH (2013). Fungal zinc metabolism and its connections to virulence. Front Cell Infect Microbiol.

[44] MacKenzie I, Rous P (1941). The experimental disclosure of latent neoplastic changes in tarred skin. J Exp Med.

[45] Guo XL, Wang LE, Du SY, Fan CL, Li L, Wang P (2003). Association of cyclooxygenase-2 expression with Hp-cagA infection in gastric cancer. World J Gastroenterol.

[46] Machado AM, Figueiredo C, Touati E, Maximo V, Sousa S, Michel V (2009). Helicobacter pylori infection induces genetic instability of nuclear and mitochondrial DNA in gastric cells. Clin Cancer Res.

[47] CarewJS HP (2002). Mitochondrial defects in cancer. Mol Cancer.

[48] Li YX, Zhang L, Simayi D, Zhang N, Tao L, Yang L (2015). Human papillomavirus infection correlates with inflammatory Stat3 signaling activity and IL-17 level in patients with colorectal cancer. PLoS ONE.

[49] Asih TS, Lenhart S, Wise S, Aryati L, Adi-Kusumo F, Hardianti MS (2015). The dynamics of HPV infection and cervical cancer cells. Bull Math Biol.

[50] Field N, Lechner M (2015). Exploring the implications of HPV infection for head and neck cancer. Sex Transm Infect.

[51] Chuang SC, La Vecchia C, Boffetta P (2009). Liver cancer: descriptive epidemiology and risk factors other than HBV and HCV infection. Cancer Lett.

[52] Beck KR, Tan SM, Lum SS, Lim LE, Krishna LK (2014). Validation of the emotion thermometers and hospital anxiety and depression scales in Singapore: screening cancer patients for distress, anxiety and depression. Asia Pac J Clin Oncol.

[53] Muirhead L (2014). Cancer risk factors among adults with serious mental illness. Am J Prev Med.

[54] Zou C, Yu S, Xu Z, Wu D, Ng CF, Yao X (2014). ERRalpha augments HIF-1 signalling by directly interacting with HIF-1alpha in normoxic and hypoxic prostate cancer cells. J Pathol.

[55] Gess B, Hofbauer KH, Deutzmann R, Kurtz A (2004). Hypoxia up-regulates triosephosphate isomerase expression via a HIF-dependent pathway. Pflugers Arch.

[56] Rotin D, Robinson B, Tannock IF (1986). Influence of hypoxia and an acidic environment on the metabolism and viability of cultured cells: potential implications for cell death in tumors. Cancer Res.

[57] DiResta GR, Lee J, Healey JH, Levchenko A, Larson SM, Arbit E (2000). Artificial lymphatic system: a new approach to reduce interstitial hypertension and increase blood flow, pH and pO2 in solid tumors. Ann Biomed Eng.

[58] Rofstad EK, Galappathi K, Mathiesen BS (2014). Tumor interstitial fluid pressure-a link between tumor hypoxia, microvascular density, and lymph node metastasis. Neoplasia.

[59] Zhong J, Rajaram N, Brizel DM, Frees AE, Ramanujam N, Batinic-Haberle I (2013). Radiation induces aerobic glycolysis through reactive oxygen species. Radiother Oncol.

[60] Kato Y, Ozawa S, Miyamoto C, Maehata Y, Suzuki A, Maeda T (2013). Acidic extracellular microenvironment and cancer. Cancer Cell Int.

[61] Kakehashi A, Wei M, Fukushima S, Wanibuchi H (2013). Oxidative stress in the carcinogenicity of chemical carcinogens. Cancers (Basel).

[62] Yin C, Qie S, Sang N (2012). Carbon source metabolism and its regulation in cancer cells. Crit Rev Eukaryot Gene Expr.

